# ACKR4 in Tumor Cells Regulates Dendritic Cell Migration to Tumor-Draining Lymph Nodes and T-Cell Priming

**DOI:** 10.3390/cancers13195021

**Published:** 2021-10-07

**Authors:** Dechen Wangmo, Prem K. Premsrirut, Ce Yuan, William S. Morris, Xianda Zhao, Subbaya Subramanian

**Affiliations:** 1 Department of Surgery, University of Minnesota Medical School, Minneapolis, MN 55455, USA; wangm005@umn.edu (D.W.); yuanx236@umn.edu (C.Y.); morr0745@umn.edu (W.S.M.); 2 Mirimus Inc., Brooklyn, NY 11226, USA; prem@mirimus.com; 3 Masonic Cancer Center, University of Minnesota, Minneapolis, MN 55455, USA; 4 Center for Immunology, University of Minnesota, Minneapolis, MN 55455, USA

**Keywords:** colorectal cancer, immune checkpoints, dendritic cells, Atypical Chemokine Receptor 4 (ACKR4), T-cell priming, immune checkpoint blockade

## Abstract

**Simple Summary:**

Our study demonstrated that Atypical Chemokine Receptor 4 (ACKR4) was downregulated in human colorectal cancer (CRC) compared with normal colon tissues. Loss of ACKR4 in human CRC was associated with a weak anti-tumor immune response. Knockdown of ACKR4 in tumor cells impairs the dendritic cell migration from the tumor to the tumor-draining lymph nodes (TdLNs), causing inadequate tumor-specific T-cell expansion and insensitivity to immune checkpoint blockades. However, loss of ACKR4 in stromal cells does not significantly affect anti-tumor immunity. In human CRC, high expression of microRNA-552 was a mechanism leading to ACKR4 downregulation. Our study revealed a novel mechanism that leads to the poor immune response in a subset of CRC and will contribute to the framework for identifying new therapies against this deadly cancer.

**Abstract:**

Colorectal cancer (CRC) is one of the most common malignancies in both morbidity and mortality. Immune checkpoint blockade (ICB) treatments have been successful in a portion of mismatch repair-deficient (dMMR) CRC patients but have failed in mismatch repair-proficient (pMMR) CRC patients. Atypical Chemokine Receptor 4 (ACKR4) is implicated in regulating dendritic cell (DC) migration. However, the roles of ACKR4 in CRC development and anti-tumor immunoregulation are not known. By analyzing human CRC tissues, transgenic animals, and genetically modified CRC cells lines, our study revealed an important function of ACKR4 in maintaining CRC immune response. Loss of ACKR4 in CRC is associated with poor immune infiltration in the tumor microenvironment. More importantly, loss of ACKR4 in CRC tumor cells, rather than stromal cells, restrains the DC migration and antigen presentation to the tumor-draining lymph nodes (TdLNs). Moreover, tumors with ACKR4 knockdown become less sensitive to immune checkpoint blockade. Finally, we identified that microRNA miR-552 negatively regulates ACKR4 expression in human CRC. Taken together, our studies identified a novel and crucial mechanism for the maintenance of the DC-mediated T-cell priming in the TdLNs. These new findings demonstrate a novel mechanism leading to immunosuppression and ICB treatment resistance in CRC.

## 1. Introduction

Colorectal cancer (CRC) is the third most commonly diagnosed malignancy and the third leading cause of cancer-related deaths in the United States [1]. By 2030, the global CRC burden is expected to increase by 60% and surpass 2.2 million new cases and 1.1 million deaths [2]. The paradigm shift in cancer treatment brought by immunotherapy has been a major scientific and clinical breakthrough. Since the first immune checkpoint blockade (ICB) therapy approval for melanoma, ICB is considered the standard of care for multiple types of cancer types, including the mismatch repair-deficient (dMMR)/microsatellite instability-high (MSI-H) CRC tumors [3]. However, not all dMMR/MSI-H CRC tumors are sensitive to ICB, and all of the mismatch repair-proficient (pMMR)/microsatellite instability-low (MSI-L)/microsatellite stability (MSS) CRC tumors are resistant to ICB [4]. Therefore, understanding the mechanisms of immunosuppression and immune therapy resistance is critical for designing novel treatments for CRC patients.

The immunogenicity of tumors is fundamental for ICB treatment. Low immunogenic tumors present a hallmark feature of sparse tumor T-cell infiltration. One of the key mechanisms involved in poor T-cell infiltration has been attributed to defects in the antigen presentation process, which significantly weakens the tumor-specific T-cell priming and precludes the T-cell mediated killing of cancer cells [5]. Dendritic cells (DCs) are the most potent antigen-presenting cells necessary to prime and activate tumor-antigen specific T-cells to induce an effective anti-tumor immune response [6,7,8]. Previous studies have shown that dysfunction of DCs caused defective antigen presentation and T-cell priming, leading to uncontrolled tumor development and ICB resistance in multiple cancers [9,10,11].

Successful antigen presentation by the DCs involves efficient migration of DCs from the tumor tissue to the regional lymph nodes. DC migration heavily depends on CCR7, a G-protein coupled receptor for two chemokines: CCL19 and CCL21 [12,13,14]. CCL21 has an extended positively charged C terminus that limits its interstitial diffusion, causing a stable gradient of CCL21 that directs the CCR7 expressing DCs from the tissue interstitium into lymphatic vessels [12,15]. On the other hand, both CCL19 and CCL21 are ligands for the atypical chemokine receptor 4 (ACKR4), a scavenging and decoy receptor that internalizes and mediates lysosomal degradation of CCL19/21 [15]. It is established that ACKR4 controls the bioavailability of CCL19/21, creating a CCL19/21 chemokine gradient that facilitates the directional migration of DCs from the non-lymphatic tissue to the draining lymph node [12,13,14,15,16]. However, the effects of ACKR4 in CRC progression and immunoregulation are largely unknown. Here, we examined the function of ACKR4 in CRC progression and anti-tumor immunity, emphasizing its role in the DC-mediated antigen presentation process and subsequent T-cell activation. Our study provides deeper insights into the immunoregulation in CRC and potentially leads to novel approaches for maximizing CRC response to ICB.

## 2. Material and Methods

### 2.1. Cell Lines and Organoids

Murine CRC cell lines MC38 and CT26 were used in the study. The source and detailed methods of cell culture are described in our previous publication [17].

### 2.2. Immunofluorescence and Histology

Human CRC tissues were fixed in 10% formalin before paraffin embedding. Sections of formalin-fixed paraffin-embedded (FFPE) tissues were deparaffinized with xylene and rehydrated with ethanol (twice in 100%, 90%, 80%, and 70%). The sections were heated in a boiling water bath with citric buffer for 12 min to retrieve antigens. Next, the sections were blocked by incubating for 30 min in 5% bovine serum albumin buffer. Tissues were incubated overnight at 4 °C with primary antibodies: anti-ACKR4 antibody (Novus, Centennial, CO, USA), anti-CD3 (Abcam, Cambridge, United Kingdom), and anti-CD11c (Abcam). The next day, the sections were washed and incubated with fluorescence-conjugated secondary antibodies (1:1000 dilution, ThermoFisher, Waltham, MA, USA) at room temperature for 1 h. After washing, the slides were mounted with ProLong Gold antifade mountant with DAPI and imaged. The researchers were blind to the ACKR4 expression level when evaluating the tumor immune infiltration. The information of primary antibodies is included in Table S1.

### 2.3. Western Blotting of ACKR4

Total protein of 40 μg was prepared from each sample and quantified by the Pierce™ BCA Protein Assay Kit (ThermoFisher). We ran the protein in sodium dodecyl sulfate–polyacrylamide (SDS) gel electrophoresis. The proteins from the gel were transferred to polyvinylidene difluoride membranes (ThermoFisher), blocked with 5% BSA, and incubated in primary antibodies overnight at 4 °C. The primary antibodies were anti-ACKR4 (Abcam) and anti-β-actin (Cell Signaling Technology, Danvers, MA, USA). The next day, the membranes were washed and incubated in peroxidase-linked anti-rabbit IgG and peroxidase-linked anti-mouse IgG for 1 h at room temperature. Pierce™ ECL Western blotting substrate (ThermoFisher) was used to image the membranes.

### 2.4. Cell Line Transfection and Transduction

We used the *ACKR4* shRNA expressing lentiviral vectors to knock down ACKR4 expression in the MC38 cell line. Briefly, 5 μg of DNA (2.5 μg of mixed shRNA expressing plasmids and 2.5 μg of pPACKH1-XL packaging vector) was mixed with 10 μL P3000^TM^ reagent in 250 μL Opti-MEM medium. The pGIPZ vector was used as the backbone of *ACKR4* shRNA expression. Then the diluted DNA was added to 250 μL Lipofectamine™ 3000 Transfection Reagent and incubated for 15 min at room temperature. The mixture was added to 5 × 10^5^ HEK293TN cells in one well of a 6-well plate. Another 500 μL Opti-MEM medium was added to make the final volume of 1000 μL. Then 24 h after the transfection, we changed the Opti-MEM medium to normal cell growth media and cultured the cells for another 24 h. Then the virus-containing media were collected and added to wild-type MC38 at different titrations. Empty shRNA vectors served as the negative control. Three days after the transduction, the transduced MC38 cells were subjected to antibiotic selection. After one week of antibiotic selection, we performed a Western blotting analysis of ACKR4 to validate the knockdown. 

### 2.5. Dendritic Cell Isolation

A Dynabeads Untouched Mouse DC Enrichment Kit (ThermoFisher) was used, and the manufacturer’s instructions were followed. Briefly, murine PBMCs were isolated from spleen, bilateral inguinal, brachial, and axillary lymph nodes by gradient centrifugation. The cells were incubated in antibody mix for 20 mins at 2 °C to 8 °C, washed, and then incubated with Depletion MyOne SA Dynabeads magnetic beads for 15 mins at 2 °C to 8 °C. The tube was placed on a magnet, and the untouched DCs in the supernatant were cultured in Iscove’s Modified Dulbecco’s Medium (IMDM) supplemented with 2000 IU/mL IL4, 2000 IU/mL granulocyte–macrophage colony-stimulating factor, and 2000 IU/mL tumor necrosis factor. All cytokines were purchased from R&D Systems (Minneapolis, MN, USA, Cat #: CDK008).

### 2.6. In Vivo DC Migration Assay

We resuspended 3 × 10^5^ freshly enriched CD45.1^+^ DCs in 50 μL PBS. We then injected them into multiple sites of MC38 subcutaneous tumors growing in C57BL/6 mouse with different ACKR4 expression (~500 mm^3^, 3 × 10^5^/tumor) by a syringe with a 30 G needle. Thirty-six hours after the injection, we sampled the tumor-draining lymph nodes (the unilateral inguinal and axillary lymph nodes). Then we isolated single cells from the tumor-draining lymph nodes (TdLNs) for detecting CD45.1^+^ DCs by FACS analysis.

### 2.7. Flow Cytometry

Mouse tumor tissues were minced into small pieces (2 × 2 × 2 mm^3^) and digested with collagenase IV (0.5 mg/mL) and deoxyribonuclease (50 units/mL) for 1 h at 37 °C. The digested tumor tissues and lymphatic tissues (TdLNs and spleens) were meshed and flushed through 70 μM and 40 μM strainers, respectively. Red blood cells were lysed by incubating the cells with red blood cell lysis buffer for 15 min and neutralizing with PBS. The cells were counted using a hemacytometer. Zombie Green fixable viability dye (BioLegend, San Diego, CA, USA) was used to count live and dead cells. All the cells were stained with primary antibody cocktails for cell surface markers. For cytoplasmic staining, cells were treated with the Cyto-Fast Fix-Perm Buffer set (BioLegend). All samples were fixed after staining. The samples were immediately analyzed in a BD FACSCanto (BD Biosciences, Franklin Lakes, NJ, USA) cytometry to prevent signal deterioration. All the data were analyzed with the FlowJo (Version 10.7.2, BD Biosciences, Franklin Lakes, NJ, USA). The information of primary antibodies is included in Table S1.

### 2.8. In Vivo T-Cell Priming Assay

We cultured 3 × 10^5^ freshly enriched DCs in 2 mL Dendritic Cell Base Media (R&D Systems) plus 10% FBS. A total of 40 μg of ovalbumin (OVA) peptides (257–264, AnaSpec) was supplied to the DC culture for a final concentration of 20 μg/mL. We also pulsed the DCs with lipopolysaccharide (LPS) (1 μg/mL) as a positive control of the DC maturation test. After 18 h of DC pulsing, we collected the DCs and injected them into multiple sites of MC38 subcutaneous tumors growing in C57BL/6 mouse with different ACKR4 expression (~300 mm^3^, 3 × 10^5^ million/tumor) by a syringe with a 30 G needle. Two weeks later, we collected the TdLNs for OVA-specific T-cell analysis.

Single cells were isolated from the TdLNs by mechanical tissue dissociation. Then, 3 × 10^5^ single cells were resuspended in 100 μL PBS with 0.1 μL Zombie Green Fixable Viability dye and incubated at room temperature for 15 min. After washing, the cells were blocked with TruStain FcX™ PLUS (0.25 µg, Biolegend) and stained with Tetramer/BV421-H-2 Kb OVA (5 μL, MBL International, Woburn, MA, USA) for 40 min at room temperature. According to the manufacture’s instruction and our preliminary experiment optimization, we used an anti-CD8 (clone KT15) antibody (MBL International) to minimize the false-positive rate of the tetramer staining. Lymphatic cells from naïve mice were used as a negative control.

### 2.9. Mouse Subcutaneous Models

The subcutaneous model was established by resuspending 5 × 10^5^ MC38 cells in 100 μL Matrigel (BD Biosciences) and injecting the tumor cell suspension into the right flank of naïve C57BL/6 mice. Following injection, using an electronic caliper, tumor growth was monitored and measured 1–2 times a week. Tumor volume was calculated using the formula

(length*width^2^)/2. (1)

### 2.10. Mouse Treatment

Mice were treated with either IgG (5 mg/kg as an anti-4-1BB control, 10 mg/kg as an anti-PD-1 control, BioXcell, Lebanon, NH, USA), anti-4-1BB agonist (5 mg/kg, clone: 3H3, BioXcell), or anti-PD-1 (10 mg/kg, clone: RMP1-14, BioXcell) on day 10, 14, and 18. All treatments were given intraperitoneally (i.p.). 

### 2.11. Quantitative PCR (qPCR) Analysis

The mirVana microRNA (miRNA) Isolation Kit (ThermoFisher Scientific) was used to extract total RNA from tumor cell lines and tissues. A total of 500 ng of total RNA was used for establishing the cDNA library with the miScript II RT Kit (Qiagen, Hilden, Germany). qRT-PCR was performed with the SYBR Green I Master kit (Roche Applied Science, Penzberg, Germany) in a LightCycler 480. The following forward primers were used: miR-552: GTTTAACCTTTTGCCTGTTGG and U6 snRNA: AAGGATGACACGCAAATTCG. The RT kit provides the universal reverse primer.

### 2.12. Enzyme-Linked Immunosorbent Assay (ELISA) for CCL21

CCL21 was quantified in tumor tissues and tumor-draining lymph nodes using an ELISA kit (Abcam). Briefly, tissue lysate samples were prepared by homogenizing tumor tissues and tumor-draining lymph nodes. We normalized the protein concentration between different samples before loading them to the experiment. The manufacturer’s instructions were followed every step.

### 2.13. Statistical Analysis

We performed all statistical analyses and graphing using GraphPad Prism software (Version 8, San Diego, CA, USA). Data were displayed as means ± SEMs. For comparison of two groups’ quantitative data, paired or unpaired Student’s *t*-tests were used. For multiple group comparison, one-way analysis of variance (ANOVA) was used followed by Bonferroni correction. Kaplan–Meier curves and log-rank tests were used to compare survival outcomes between groups. We used the chi-square test to compare two variables in a contingency table to see if they were related. A two-tail *p*-value of less than 0.05 was considered statistically significant.

## 3. Results

### 3.1. ACKR4 Is Downregulated in CRC Compared with Normal Colon

**Figure 1:**
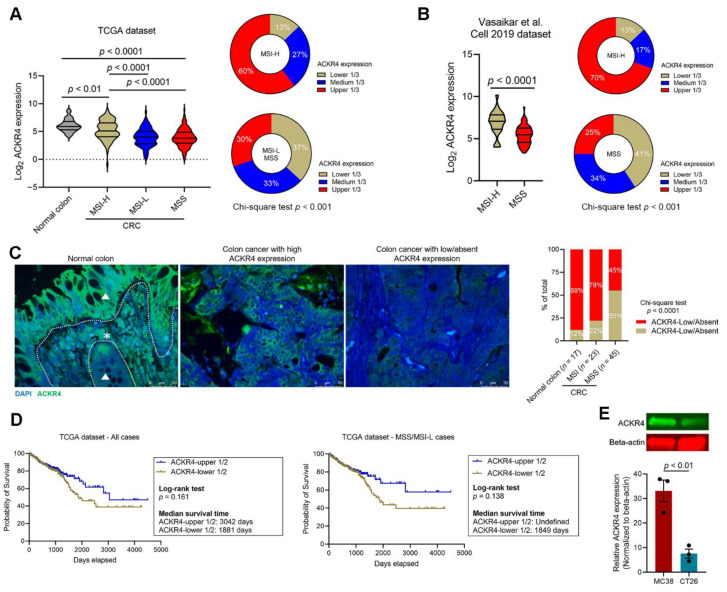
ACKR4 expression in human CRC tissue sample and cell lines. (**A**) *ACKR4* mRNA expression in the TCGA CRC dataset. The normal colon tissues had a higher ACKR4 expression level than the CRC tissues. The MSI-H subtype tumors had an elevated ACKR4 expression level compared to the MSI-L and MSS subtype tumors. (**B**) The *ACKR4* transcript levels in another independent Vasaikar et al. [18] dataset. (**C**) Immunofluorescence staining of ACKR4 in human normal colon tissues (*n* = 17) and CRC tumor tissues (*n* = 23 for MSI tumors and *n* = 45 for MSS tumors). The representative micrographs showed the low and high ACKR4 expression cases (The white dot line indicates the border of epithelium and stroma. The star indicates tumor stroma. The triangle indicates epithelium). (**D**) The overall survival curve of CRC patients with high or low ACKR4 expression (for the TCGA dataset, the median value of ACKR4 expression was used as the cut-off point). The comparisons were made in all CRC cases (left panel) or MSS and MSI-L cases (right panel; undefined means more than 50% of patients survive at the follow-up). (**E**) Western blotting analysis of ACKR4 expression in mouse CRC cell lines (*n* = 3). The ACKR4 expression level was normalized to the β-actin levels. (For more than two group statistical analyses, the uppermost *p*-value indicates the ANOVA-analysis, and other *p*-values indicate the posthoc analysis between two specific groups. TCGA: The Cancer Genome Atlas, MSS: Microsatellite stability, MSI-L: Microsatellite instability-low, MSI-H: Microsatellite instability-high, ACKR4: Atypical Chemokine Receptor 4, CRC: Colrectal cancer, ANOVA: Analysis of variance). Detailed information about the Western blotting can be found in Figure S3.

To investigate the immunoregulatory role of ACKR4 in CRC, we first evaluated the ACKR4 expression in CRC and normal colon tissues. Analysis of the CRC dataset in the The Cancer Genome Atlas (TCGA) and another independent dataset reported by Vasaikar et al. [18] showed that ACKR4 expression was lower in CRC than in normal colon tissues (Figure 1A,B). Further stratification of the CRC cases based on the MSI/MSS statuses indicated that ACKR4 expression was lower in MSS/MSI-L tumors than the MSI-H tumors (Figure 1A,B). The immunofluorescence staining on sections of 68 human CRC and 17 normal colon tissues revealed that 88% of normal colon tissues and 78% of MSI-CRC tissues have abundant ACKR4 expression. In contrast, only 45% of MSS-CRC tissues have a similar ACKR4 level. These data confirmed the downregulation of ACKR4 in CRC tissues, especially in the MSS subtype (Figure 1C). Next, we evaluated the prognostic significance of ACKR4 in the TCGA cohort (Figure 1D). Although not statistically significant, patients with higher ACKR4 expression are more likely to have a longer median survival time than patients with lower ACKR4 expression (Figure 1D). To control the influence of MSS/MSI status on the survival benefit, we removed the MSI-H cases and performed a subgroup analysis with the MSS and MSI-L samples. Again, the ACKR4 high cases are more likely to have a better prognosis (Figure 1D). Finally, we determined the ACKR4 level in the mouse CRC cell lines, which are widely used in immunological studies. Notably, the mouse CRC cell line MC38 (MSI phenotype) had significantly higher ACKR4 expression than the CT26 cell line (MSS phenotype) (Figure 1E).

### 3.2. Knockdown of ACKR4 in Tumor Cells but Not the Host Tissues Accelerate Tumor Growth

**Figure 2:**
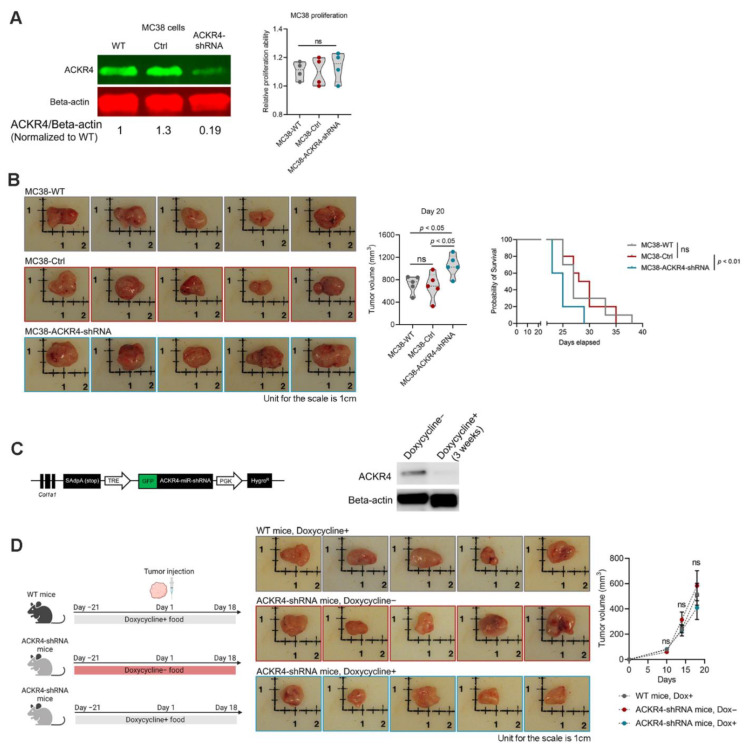
ACKR4 expression and tumor development. (**A**) Western blot analysis of ACKR4 knockdown in MC38 cell line (The grey area defines the data distribution. The dot lines in the violin plots indicate quartiles). (**B**) Knockdown of ACKR4 accelerated MC38 tumor growth in naïve C57BL/6J mice (*n* = 5 for tumor growth analysis and *n* = 10 for survival analysis). (**C**) The induction and confirmation of ACKR4 knockdown in transgenic mice. Doxycycline treatment for 3 weeks significantly reduced ACKR4 expression in the mouse skin and subcutaneous connective tissue. (**D**) Knockdown of ACKR4 in the host mice did not significantly affect MC38 tumor growth (*n* = 5). (For more than two group statistical analyses, the uppermost *p*-value indicates the ANOVA-analysis, and other *p*-values indicate the posthoc analysis between two specific groups. WT: Wild type, Ctrl: Control, shRNA: Short hairpin RNA, Dox: Doxycycline, Col1a1: Collagen, type I, alpha 1, GFP: Green fluorescent protein, Hygro^R^: Hygromycin resistance, PGK: Phosphoglycerate kinase, TRE: Tetracycline response element, ns: No significance, ANOVA: Analysis of variance). Detailed information about the Western blotting can be found in Figures S4 and S5.

Next, we sought to determine the impact of ACKR4 downregulation in CRC development. Using the vector-based short hairpin RNA (shRNA) interference technology, we knocked down ACKR4 expression in the MC38 cell line, which has relatively high endogenous ACKR4 expression ((Figure 1E and (Figure 2A). Knockdown of ACKR4 did not significantly influence the MC38 cell proliferation in vitro (Figure 2A). We then injected the MC38 cells subcutaneously into naïve C57BL/6 mice. Notably, the knockdown of ACKR4 in the tumor cells accelerated tumor growth in vivo (Figure 2B). To see whether the ACKR4 level in the host tissue also affects tumor development, we established a conditional ACKR4 knockdown mouse model (Figure 2C). We knocked down ACKR4 expression in the host mice by doxycycline treatment before MC38 tumor cell injection. However, the knockdown of ACKR4 in host tissue did not significantly alter the tumor development (Figure 2D). Our results indicated that ACKR4 of tumor cells is more competent in regulating tumor growth than the host ACKR4.

### 3.3. Loss of ACKR4 Reduces Tumor T-Cell Infiltration

**Figure 3:**
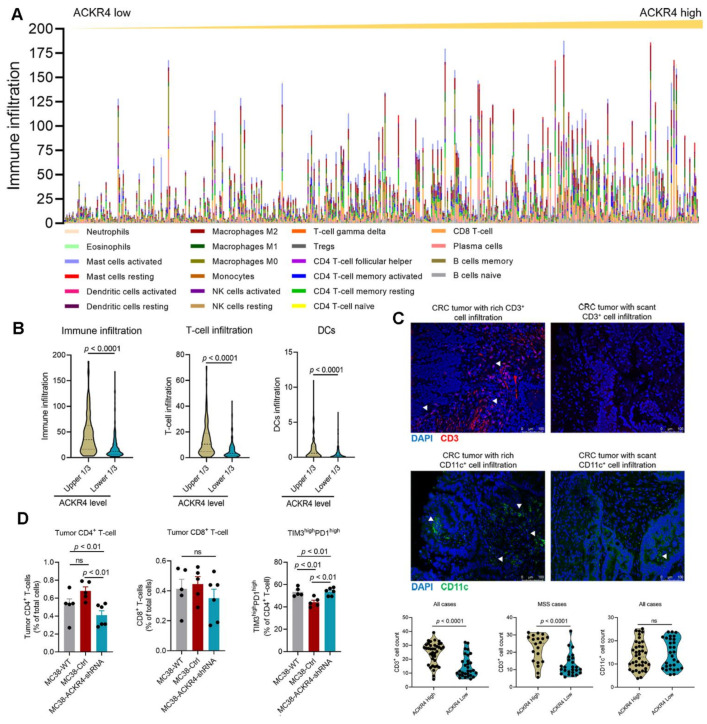
ACKR4 expression and tumor immune cell infiltration. (**A,B**) The immune profiles of CRC cases in the TCGA dataset generated by the CIBERSORT. Elevated ACKR4 expression is associated with higher total immune cells, T-cells, and DC infiltration (The dot lines in the violin plots indicate quartiles). (**C**) Immunofluorescence analysis of CD3 and CD11c on human CRC tissues. High ACKR4 expression was associated with high T-cell (CD3^+^) but not dendritic cell (CD11c^+^) infiltration (*n* = 68, the triangles indicate positive staining, the dot lines in the violin plots indicate quartiles). (**D**) FACS analysis on tumor-infiltrating T-cells on MC38 tumor models. ACKR4 knockdown MC38 tumors had fewer CD4^+^ T-cells in their tumor microenvironment. The percentage of exhausted CD4^+^ T-cells was higher in the ACKR4 knockdown tumors than in the controls (*n* = 5–6). (For more than two group statistical analyses, the uppermost *p*-value indicates the ANOVA-analysis, and other *p*-values indicate the posthoc analysis between two specific groups. DCs: Dendritic cells, CD8: Cluster of differentiation 8, CD4: Cluster of differentiation 4, CD3: Cluster of differentiation 3, CD11c: Cluster of differentiation 11c, WT: Wild type, Ctrl: Control, shRNA: Short hairpin RNA, TCGA: The Cancer Genome Atlas, CRC: Colrectal cancer, Treg: Regulatory T-cell, TIM3: T-cell immunoglobulin domain and mucin domain 3, PD1: Programmed cell death protein 1, MSS: Microsatellite stability, DAPI: 4′,6-diamidino-2-phenylindole, ANOVA: Analysis of variance, ns: No significance).

To study whether the tumor growth caused by ACKR4 knockdown was associated with anti-tumor immunity, we analyzed the tumor immune infiltration in the TCGA CRC dataset by the CIBERSORT algorithm (Figure 3A,B and Figure S1). Tumors with higher ACKR4 expression had elevated immune cell infiltration, including the total T-cells, CD8^+^ T-cells, CD4^+^ T-cells, regulatory T-cells (Treg), and total DCs, compared to tumors with lower ACKR4 expression (Figure 3B and Figure S1A–C). Higher ACKR4 expression was also associated with more total NK cells, B-cells, and polarized macrophages in the tumor microenvironment (Figure S1D–H). Histological analysis on human CRC tissues confirmed that ACKR4 high-expressing tumors are associated with a higher number of tumor-infiltrating T-cells (Figure 3C). However, there was no difference in DC infiltration between the ACKR4-high and -low groups (Figure 3C). Next, we investigated the immune infiltration in ACKR4 knockdown tumor models (Figure S2A,B). Our results show that ACKR4 knockdown tumors have fewer CD4^+^ T-cells but a higher proportion of exhausted CD4^+^ T-cells in their tumor microenvironment than the control group (Figure 3D). However, the frequencies of tumor-infiltrating CD8^+^ T-cells and DCs are not influenced by ACKR4 expression (Figure 3D and Figure S2C). The ACKR4 level in tumor cells also does not systemically change the frequency and function of immune cells in the tumor-draining lymph nodes (Figure S2D).

### 3.4. Loss of ACKR4 Impairs DC Migration to Tumor-Draining Lymph Nodes and Tumor-Specific T-Cell Expansion

**Figure 4:**
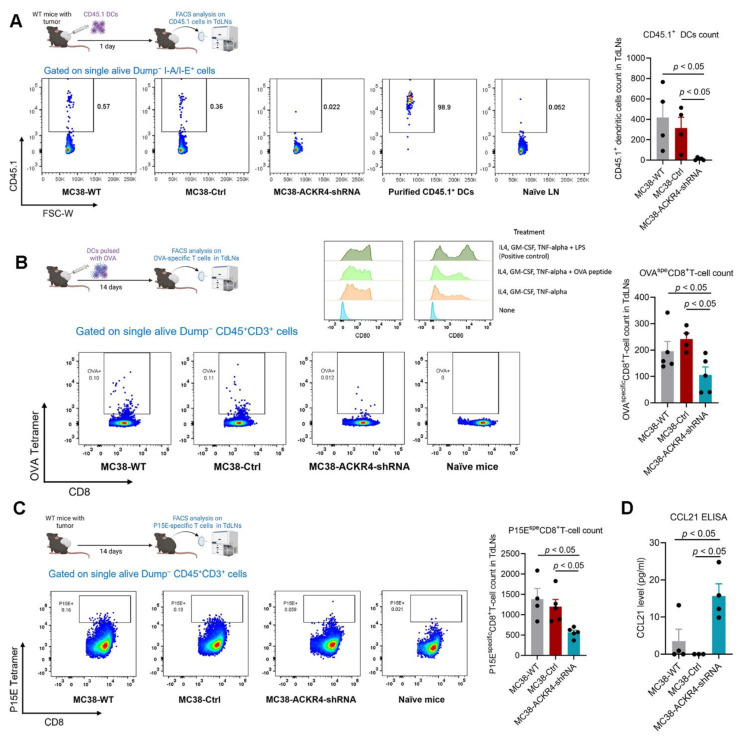
ACKR4 expression and DC migration and T-cell priming. (**A**) Enriched CD45.1^+^ DCs were injected into the MC38 tumor and analyzed in TdLNs 1 day post-injection. ACKR4 knockdown in MC38 tumor cells impaired DC migration from the tumor to the TdLNs (*n* = 4). (**B**) DCs loaded with OVA antigens were injected into the MC38 tumor microenvironment, and the OVA-specific CD8^+^ T-cells were analyzed in the TdLNs. ACKR4 knockdown in MC38 tumor cells impaired DC mediated T-cell priming (*n* = 4–5). The histogram shows CD80 and CD86 expression on DCs used in the study. (**C**) P15E (a tumor-associated antigen in MC38 cells)-specific CD8^+^ T-cell counts in TdLNs of MC38 tumors with various ACKR4 expression levels (*n* = 4–5). (**D**) CCL21 quantification in MC38 tumors with different ACKR4 expression levels (*n* = 3–4). (For more than two group statistical analyses, the uppermost *p*-value indicates the ANOVA-analysis, and other *p*-values indicate the posthoc analysis between two specific groups. DCs: Dendritic cells, OVA: Ovalbumin, CD8: Cluster of differentiation 8, CD3: Cluster of differentiation 3, CD80: Cluster of differentiation 80, CD86: Cluster of differentiation 86, CCL21: Chemokine (C-C motif) ligand 21, FACS: Fluorescence-activated cell sorting, TdLNs: Tumor-draining lymph nodes, P15E: Murine leukemia virus envelope protein P15E, FSC-W: Forward light scatter width, CD45.1: Cluster of differentiation 45.1, CD45: Cluster of differentiation 45, WT: Wild type, Ctrl: Control, shRNA: Short hairpin RNA, IL4: Interleukin 4, GM-CSF: Granulocyte-macrophage colony-stimulating factor, TNF: Tumor necrosis factor, LPS: Lipopolysaccharides, Spe: Specific, ANOVA: Analysis of variance).

Since ACKR4 regulates the CCL21 chemokine gradient [12], we hypothesized that loss of ACKR4 in tumor tissue would increase the CCL21 levels in the tumor microenvironment. An increase of CCL21 in the tumor tissue will potentially impede DC migration, mediated by the CCL21 chemokine gradient between the tumor tissue and the tumor-draining lymph nodes (TdLNs). To validate this hypothesis, we injected the CD45.1^+^ DCs into tumors with wild-type or knocked-down ACKR4 expression. We then analyzed the amount of CD45.1^+^ DCs in the TdLNs. Notably, DCs in the wild-type and control tumors are more likely to migrate to the TdLNs than the DCs in the ACKR4 knockdown tumors (Figure 4A). To observe whether the reduction of DC migration would cause the impaired tumor-specific T-cell priming in the TdLNs, we tested for the antigen-specific T-cells in the TdLNs. We first pulsed the DCs with the ovalbumin (OVA) antigen and then injected them into the tumors. We confirmed the DCs we used expressing DC maturation markers, CD80 and CD86 (Figure 4B). We analyzed the OVA-specific CD8^+^ T-cells in the TdLNs and found that AKCR4 knockdown in the tumor significantly reduced the DC mediated antigen-specific T-cell priming in the TdLNs (Figure 4B). We also confirmed the finding with the endogenous tumor antigen (Figure 4C). Finally, we determined that the CCL21 level in the ACKR4 knockdown tumor tissues was significantly higher than in the wild-type and control groups (Figure 4D).

### 3.5. Loss of ACKR4 Weakens Tumor Response to Immune Checkpoint Blockade

**Figure 5:**
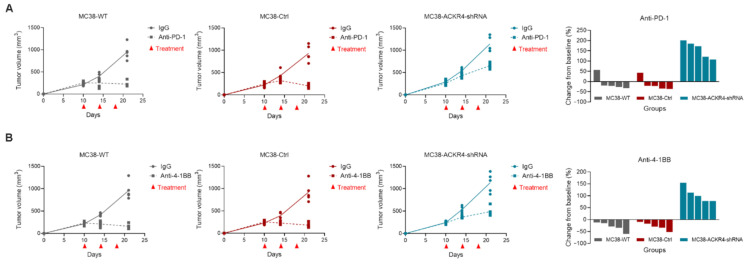
Immunotherapy response on MC38 tumors with different ACKR4 expression levels. (**A**) The mice were treated by anti-PD-1 on days 10, 14, and 18. The waterfall plot shows the individual tumor volume change post-treatment. The response of the ACKR4 knockdown group to anti-PD-1 treatment was worse than that of the other groups. (**B**) The anti-4-1BB agonist treatment showed similar results to the anti-PD-1 treatment. (WT: Wild type, Ctrl: Control, shRNA: Short hairpin RNA, PD-1: Programmed cell death protein 1, 4-1BB: CD137/Tumor necrosis factor receptor superfamily 9, IgG: Immunoglobulin G, ANOVA: Analysis of variance).

Because ACKR4 knockdown reduces the tumor infiltrating T-cells and DC mediated tumor-specific T-cell expansion in the TdLNs (Figure 3 and Figure 4), we next evaluated whether ACKR4 knockdown affects the tumor response to immune checkpoint blockade. We treated the wild-type, control, and ACKR4 knockdown tumors with anti-PD-1 or anti-4-1BB antibodies. The ACKR4 knockdown tumors were less sensitive to anti-PD-1 or anti-4-1BB treatments than wild-type and control tumors (Figure 5). This result suggested that loss of ACKR4 could be implicated in the immune checkpoint blockade resistance in CRC.

### 3.6. MicroRNA miR-552 Downregulates ACKR4 in CRC

**Figure 6:**
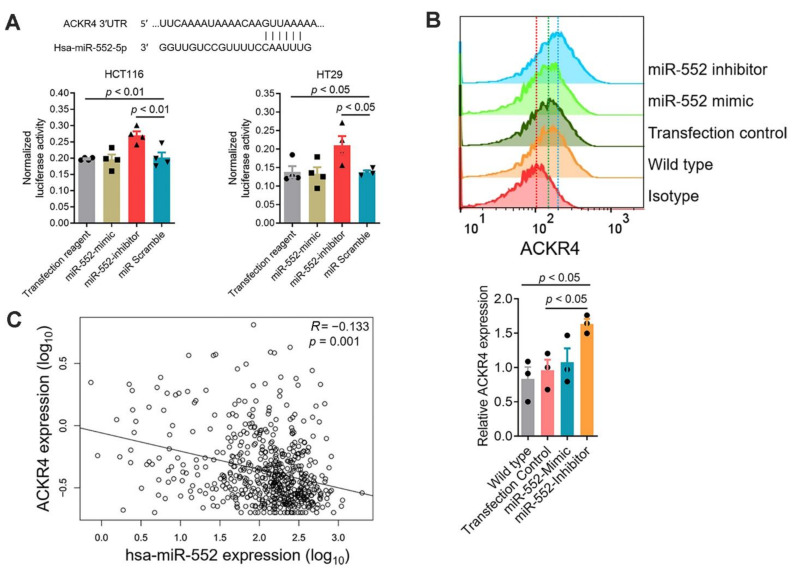
miR-552 downregulates ACKR4 expression in CRC tumors. (**A**) The sequence match between the miR-552 and the *ACKR4* 3′-untranslated region (UTR). Dual-luciferase assay confirmed that miR-552 binds to the 3′-UTR of *ACKR4* (*n* = 4). (**B**) miR-552 inhibitors enhanced ACKR4 expression in HCT116 cells (*n* = 3, the red vertical dot line indicates the isotype group’s mean signal intensity, the green dot line indicates the transfection control group’s mean signal intensity, and the blue vertical dot line indicates the miR-552 inhibitor group’s mean signal intensity). (**C**) A negative correlation between *ACKR4* and miR-552 in the TCGA colorectal cancer dataset (The black line is the regression line). (For more than two group statistical analyses, the uppermost *p*-value indicates the ANOVA-analysis, and other *p*-values indicate the posthoc analysis between two specific groups. miR: MicroRNA, Hsa: Homo sapiens, TCGA: The Cancer Genome Atlas, ANOVA: Analysis of variance).

Our previous microRNA (miRNA) expression profiling analysis had shown that miR-552 is highly expressed in MSS-CRC, which does not respond to immune checkpoint blockade [19]. Further sequence match analysis showed that miR-552 potentially binds to human ACKR4 transcript and subsequently downregulates ACKR4 expression (Figure 6A). Our dual luciferase assay and flow cytometry analysis confirmed the effects of miR-552 on ACKR4 downregulation in human CRC cell lines (Figure 6A,B). Analysis of the TCGA-CRC dataset further confirmed the negative correlation between miR-552 and ACKR4 (Figure 6C).

## 4. Discussion

Investigating the regulatory mechanism of tumor immunity is essential to alleviate drug resistance and improve the effect of immunotherapy [20,21]. As the key cell type in the process of antigen presentation, DCs and their function are closely associated with the intensity of tumor immunity [9,10,11]. The CCR7 expressed on DCs and the CCL19/21 gradient in the interstitial compartment largely regulates DC migration [12,13]. ACKR4 shapes the CCL19/21 gradient between the non-lymphatic and lymphatic tissues by scavenging both the soluble and immobilized CCL19/CCL21 [12,13]. In breast cancer, nasopharyngeal cancer, liver cancer, and cervical cancer, ACKR4 negatively regulates tumor growth and metastasis, implying a protective role in tumorigenesis [22,23,24,25]. However, the role of ACKR4 in tumor immunogenicity and overall anti-tumor immunity of CRC has not been determined.

Our study first evaluated the expression of ACKR4 in human normal colon and CRC tissues and revealed that ACKR4 was downregulated in CRC. This result corroborates a recent study showing that villous colon adenomas have less ACKR4 expression than the normal colon tissues [26]. Further analysis indicated that the MSI-H CRC had relatively higher expression of ACKR4 than the MSI-L/MSS CRC samples. These data showed the correlation between ACKR4 expression and CRC progression, providing the cornerstone for further studying the implications of ACKR4 in CRC pathobiology.

A key question is whether AKCR4 of tumor cells or ACKR4 of tumor-associated stromal cells affects tumor growth. Taking advantage of the inducible ACKR4 knockdown mice model, we were able to allow the mice to mature with intact ACKR4 expression and selectively downregulate the ACKR4 expression in the host right before and during wild-type MC38 tumor development. In another model, we knocked down ACKR4 in MC38 cells, which have a relatively high endogenous ACKR4 expression, and injected those cells into wild-type mice. Notably, ACKR4 knockdown in MC38 cells significantly accelerated tumor growth. However, ACKR4 expression in the stromal cells did not affect tumor growth. These results highlighted the distinct functions of ACKR4 in tumor cell and stromal cell compartments. Our data are distinctive from the previous study showing that ACKR4 knockout mice delayed E0771 mammary tumor growth [27]. These differences may be attributed to the different tumor cell lines tested. Although there are still controversies, permanent germline ACKR4 knockout may cause abnormalities in immune organ development [28,29,30]. This might be another reason why our results from inducible ACKR4 knockdown mice are different from embryonic ACKR4 knockout mice.

DCs have been identified as the most potent antigen-presenting cells in tumor antigen presentation and T-cell priming [6,9,10,11]. ACKR4, a decoy receptor that binds and degrades CCR7 ligands CCL19/CCL21, regulates DC migration from skin to the regional lymph nodes [12,13]. However, whether similar effects exist in tumor conditions remains unknown. Our work demonstrated that in the case of ACKR4 knockdown, tumor-infiltrating DCs are less likely to migrate towards TdLNs, causing a weak tumor-specific T-cell expansion in TdLNs. Consequently, the intensity of anti-tumor immunity and response to ICB was significantly restricted by ACKR4 downregulation. These data support our previous work showing that the immune response that occurs in TdLNs is extremely critical for initiating anti-tumor immunity [31]. In addition, our study also indicates that miR-552 negatively regulates ACKR4, and blocking the function of miR-552 increases ACKR4 expression in human CRC cell lines. Those results provided a potential target to rescue the ACKR4 expression in tumors.

Although our work has efficiently demonstrated the ACKR4 function in anti-tumor immunity, a few limitations remain. First, we did not investigate whether the ACKR4 function is dependent on the CCR7. However, it is the next step to determine if the ACKR4-mediated immunoregulation relies entirely on the CCR7 signaling or other pathways. Moreover, our work is restricted to the MC38 cell line in wild-type and ACKR4 knockdown mice. Due to technical difficulties, we could not overexpress ACKR4 in another widely used CRC cell line, CT26, which has a low ACKR4 expression. Further work with additional preclinical models are needed to confirm the conserved mechanism of ACKR4 mediated immunoregulation.

## 5. Conclusions

In conclusion, our work indicated that loss of ACKR4 in CRC is associated with poor anti-tumor immune infiltration. Mechanistically, the knockdown of ACKR4 in tumor cells restricts DC migration from tumor tissue to the tumor draining lymph nodes, thus impairing the tumor-specific T-cell priming and response to ICB. These data, collectively, describe a novel immunosuppressive mechanism and increase our understanding of how intrinsic tumor factors affect DC-mediated immune response in CRC.

## Data Availability

Data available on request from the corresponding author due to privacy.

## Supplementary Materials

The following are available online at https://www.mdpi.com/article/10.3390/cancers13195021/s1, Figure S1: ACKR4 expression and tumor immune infiltration in TCGA CRC dataset, Figure S2: ACKR4 expression and tumor immune infiltration, Figure S3: Full Western blot images for Figure 1E, Figure S4: Full Western blot images for Figure 2A, Figure S5: Full Western blot images for Figure 2C, Table S1: Key resources.
