# Supplementary material for: ACKR4 in Tumor Cells Regulates Dendritic Cell Migration to Tumor-Draining Lymph Nodes and T-Cell Priming
Source: Cancers (Basel). 2021 Oct 7;13(19):5021. doi: 10.3390/cancers13195021 (PMC8507805; doi:10.3390/cancers13195021)
Supplement: Supplementary file 1 [file cancers-13-05021-s001.zip › cancers-1377799-supplementary-final/cancers-1377799-supplementary-final.pdf]

*Supplementary Materials*

# ACKR4 in Tumor Cells Regulates Dendritic Cell Migration to Tumor-Draining Lymph Nodes and T-Cell Priming

**Dechen Wangmo, Prem K. Premsrirut, Ce Yuan, William S. Morris, Xianda Zhao and Subbaya Subramanian**

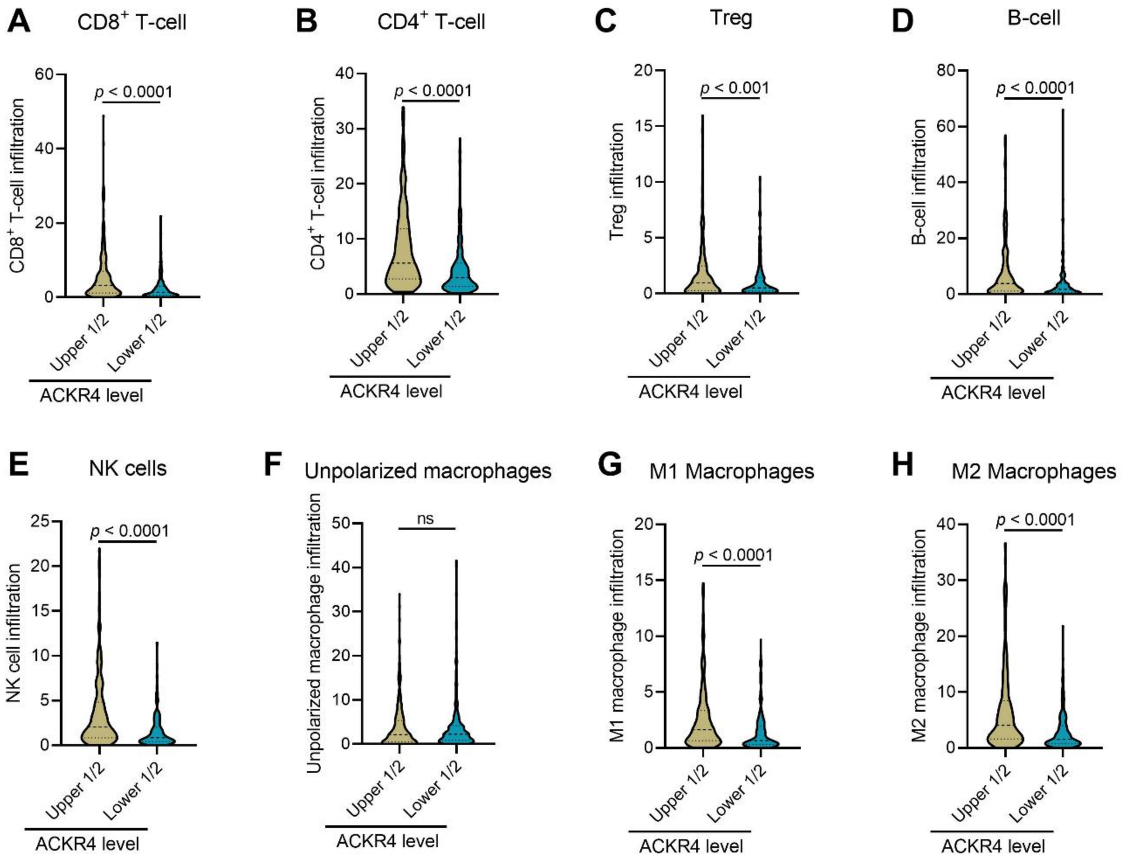


**Figure S1.** ACKR4 expression and tumor immune infiltration in TCGA CRC dataset. (**A**–**H**) Statistical analyses were performed to compare the amount of major immune cell subtypes between ACKR4 high expressing and low expressing tumors. The data were related to Figure 3A. (The dot lines in the violin plots indicate quartiles. CD8: Cluster of differentiation 8, CD4: Cluster of differentiation 4, Treg: Regulatory T-cell, NK: Natural killer, ns: No significance).

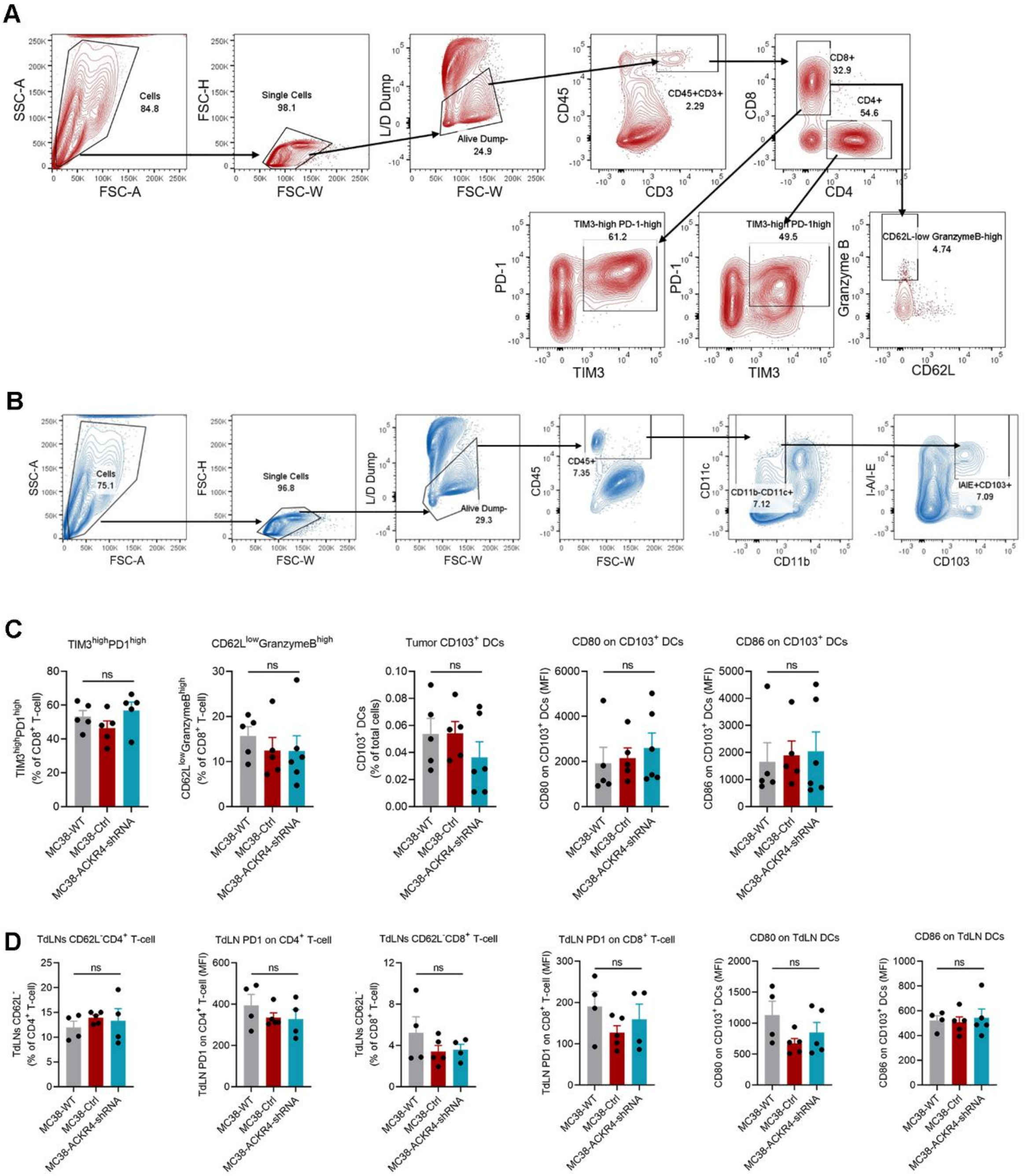


**Figure S2.** ACKR4 expression and tumor immune infiltration. (**A,B**) The gating strategy for tumor-infiltrating T cells and CD103$^{+}$ dendritic cells. (**C**) ACKR4 knockdown in MC38 tumors did not significantly change the frequencies of exhausted and activated CD8$^{+}$ T cells and CD103$^{+}$ DCs in the tumor microenvironment. The ACKR4 expression on tumor cells did not alter the CD80 and CD86 expression on the CD103$^{+}$ DCs ($n$ = 5–6). (**D**) The ACKR4 expression on tumor cells did not change the functional status of the general T cells and DCs in the TdLNs ($n$ = 4–5). (DCs: Dendritic cells, CD8: Cluster of differentiation 8, CD4: Cluster of differentiation 4, CD3: Cluster of differentiation 3, CD11c: Cluster of differentiation 11c, CD103: Cluster of differentiation 103, CD62L: Cluster of differentiation 62L/L-selectin, WT: Wild type, Ctrl: Control, shRNA: Short hairpin RNA, TdLN: Tumor-draining lymph node, TIM3: T-cell immunoglobulin domain and mucin domain 3, PD1: Programmed cell death protein 1, SSC-A: Side scatter area, FSC-W: Forward light scatter width, FSC-A: Forward light scatter area, FSC-H: Forward light scatter height, L/D: Live or dead, ns: No significance).

Exposures on the same membrane

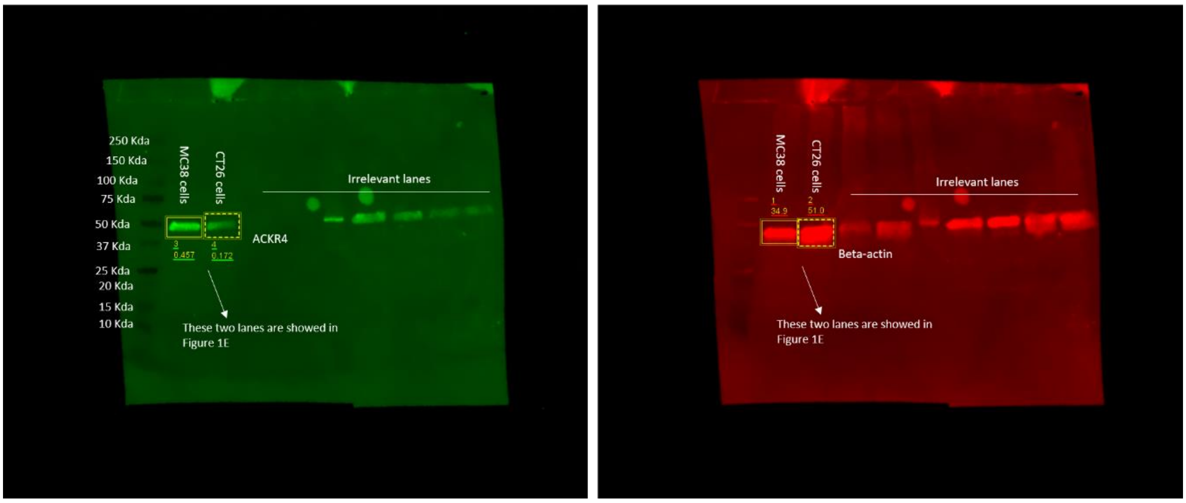


**Figure S3.** Full Western blot images for Figure 1E.

Exposures on the same membrane

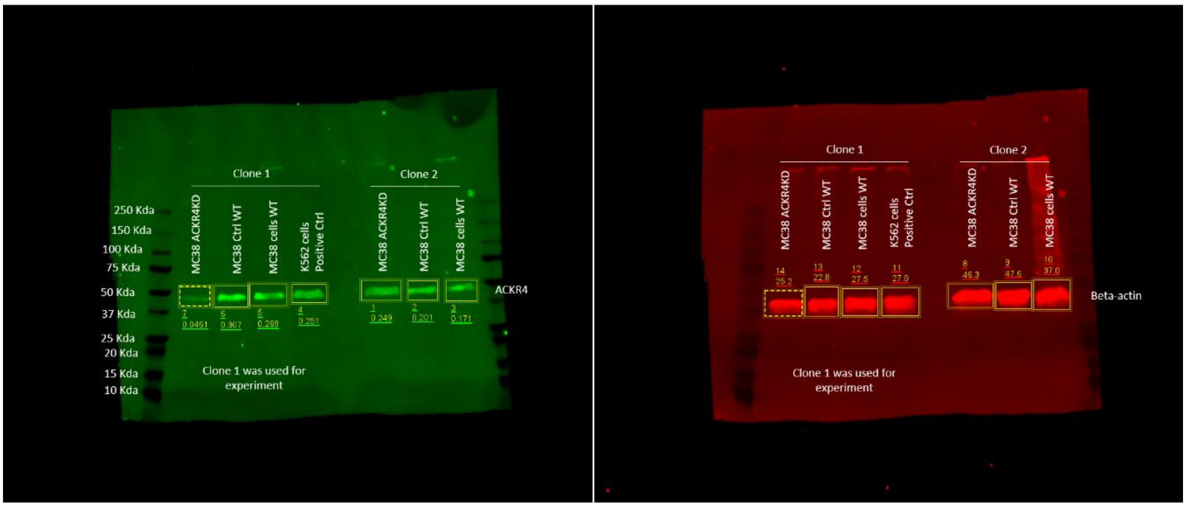


**Figure S4.** Full Western blot images for Figure 2A.

Exposures on the same membrane

Chemical channel: Beta-actin; 800 nm channel: Ladder

Chemical channel: ACKR4; 800 nm channel: Ladder

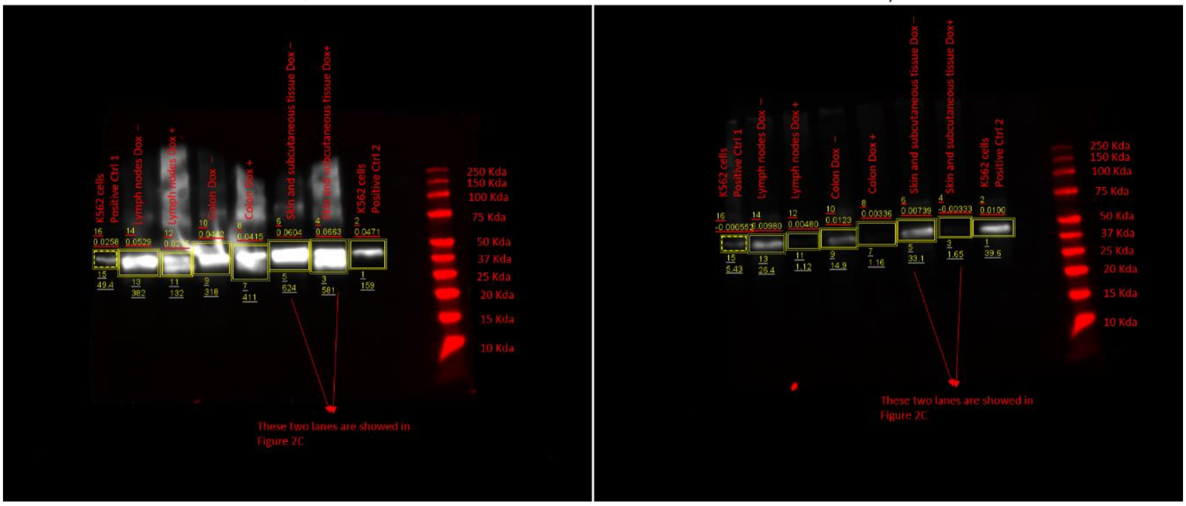


**Figure S5.** Full Western blot images for Figure 2C.

**Table S1.** Key resources.

| Reagent for Immunoassay | Source | Identifier |
|---|---|---|
| Anti-human/mouse CD3 | Abcam (Cambridge, United Kingdom) | Cat #: ab11089 |
| Anti-human CD11c | Abcam (Cambridge, United Kingdom) | Cat #: ab52632 |
| Anti-human CD11c | Abcam (Cambridge, United Kingdom) | Cat #: ab212508 |
| Anti-human ACKR4 | Novus (Centennial, CO, USA) | Cat #: NB100-705 |
| Anti-human/mouse ACKR4 | Abcam (Cambridge, United Kingdom) | Cat #: ab32564 |
| Anti-human/mouse β-actin | Novus (Centennial, CO, USA) | Cat #: NB600-501 |
| Goat anti-rat secondary antibody, Alexa Fluor 568 | Invitrogen (Waltham, MA, USA) | Cat #: A11077 |
| Goat anti-rabbit secondary antibody, Alexa Fluor 488 | Invitrogen (Waltham, MA, USA) | Cat #: A11008 |
| Goat anti-mouse secondary antibody, Alexa Fluor 488 | Invitrogen (Waltham, MA, USA) | Cat #: A11001 |
| Donkey anti-goat secondary antibody, Alexa Fluor 488 | Invitrogen (Waltham, MA, USA) | Cat #: A11055 |
| Anti-human HLA-DR-PE (L243) | BioLegend (San Diego, CA, USA) | Cat #: 307606 |
| Anti-human CD14-Pacific Blue (63D3) | BioLegend (San Diego, CA, USA) | Cat #: 367122 |
| Anti-human CD1a-FITC (HI149) | BioLegend (San Diego, CA, USA) | Cat #: 300104 |
| Anti-human CD80-BV 510 (2D10) | BioLegend (San Diego, CA, USA) | Cat #: 305234 |
| Anti-human CD45-APC/Cy7 (HI30) | BioLegend (San Diego, CA, USA) | Cat #: 304014 |
| Anti-human CD11c-APC (3.9) | BioLegend (San Diego, CA, USA) | Cat #: 301614 |
| Anti-mouse CD4-BV510 (GK1.5) | BioLegend (San Diego, CA, USA) | Cat #: 100449 |
| Anti-mouse/human CD11b-FITC (M1/70) | BioLegend (San Diego, CA, USA) | Cat #: 101205 |
| Anti-mouse NK-1.1-FITC (PK136) | BioLegend (San Diego, CA, USA) | Cat #: 108705 |
| Anti-mouse CD19-FITC (6D5) | BioLegend (San Diego, CA, USA) | Cat #: 115505 |
| Anti-mouse PD-1-PerCP/Cy5.5 (29F.1A12) | BioLegend (San Diego, CA, USA) | Cat #: 135208 |
| Anti-mouse CD3-PE/Cy7 (17A2) | BioLegend (San Diego, CA, USA) | Cat #: 100220 |
| Anti-mouse CD45-APC/Cy7 (30-F11) | BioLegend (San Diego, CA, USA) | Cat #: 103115 |
| Anti-mouse CD366 (Tim-3)-BV421 (RMT3-23) | BioLegend (San Diego, CA, USA) | Cat #: 119723 |
| Anti-mouse CD4-BV510 (GK1.5) | BioLegend (San Diego, CA, USA) | Cat #: 100449 |
| Anti-mouse CD3-FITC (17A2) | BioLegend (San Diego, CA, USA) | Cat #: 100204 |
| Anti-mouse CD86-PE (GL-1) | BioLegend (San Diego, CA, USA) | Cat #: 105007 |
| Anti-mouse CD11c-PerCP/Cy5.5 (N418) | BioLegend (San Diego, CA, USA) | Cat #: 117328 |
| Anti-mouse CD80-PE/Cy7 (16-10A1) | BioLegend (San Diego, CA, USA) | Cat #: 104734 |
| Anti-mouse/human CD11b-APC (M1/70) | BioLegend (San Diego, CA, USA) | Cat #: 101212 |
| Anti-mouse I-A/I-E-APC/Cy7 (M5/114.15.2) | BioLegend (San Diego, CA, USA) | Cat #: 107628 |
| Anti-mouse CD45.1-BV421 (A20) | BioLegend (San Diego, CA, USA) | Cat #: 110732 |
| Anti-CD8 (mouse) mAb-PE (monoclonal antibody) | MBL International (Woburn, MA, USA) | Cat #: D271-5 |
| Anti-mouse CD62L-PE/Cy7 (MEL-14) | BioLegend (San Diego, CA, USA) | Cat #: 104418 |
| Anti-mouse CD8a-APC/Cy7 (53-6.7) | BioLegend (San Diego, CA, USA) | Cat #: 100714 |
| Anti-mouse CD45-Pacific Blue (30-F11) | BioLegend (San Diego, CA, USA) | Cat #: 103126 |
| Anti-mouse CD103-Pacific Blue (2E7) | BioLegend (San Diego, CA, USA) | Cat #: 121418 |
| Anti-mouse/human CD11b-APC (M1/70) | BioLegend (San Diego, CA, USA) | Cat #: 101212 |
| Anti-mouse CD45-BV510 (30-F11) | BioLegend (San Diego, CA, USA) | Cat #: 103138 |
| Anti-human/mouse granzyme B-AF647 (GB11) | BioLegend (San Diego, CA, USA) | Cat #: 515406 |
| Anti-mouse IFN-γ-PerCP/Cy5.5 (XMG1.2) | BioLegend (San Diego, CA, USA) | Cat #: 505822 |
| Anti-mouse CD3-PE (17A2) | BioLegend (San Diego, CA, USA) | Cat #: 100206 |
| Anti-mouse CD45-PE (30-F11) | BioLegend (San Diego, CA, USA) | Cat #: 103106 |
| Anti-mouse CD3-APC (17A2) | BioLegend (San Diego, CA, USA) | Cat #: 100236 |
| Tetramer/BV421–H-2 Kb OVA (SIINFEKL) | MBL International (Woburn, MA, USA) | Cat #: TB-5001-4 |
| T-Select I-Ab OVA 323-339 Tetramer-APC | MBL International (Woburn, MA, USA) | Cat #: TS-M710-2 |
| H-2Kb MuLV p15E Tetramer-KSPWFTTL-APC | MBL International (Woburn, MA, USA) | Cat #: TB-M507-2 |
| Rat IgG1, κ Isotype Ctrl-Pacific Blue (RTK2071) | BioLegend (San Diego, CA, USA) | Cat #: 400419 |
| Rat IgG1, κ Isotype Ctrl-PE/Cy7 (RTK2071) | BioLegend (San Diego, CA, USA) | Cat #: 400415 |
| Mouse IgG1, κ Isotype Ctrl-PE/Cy7 (MOPC-21) | BioLegend (San Diego, CA, USA) | Cat #: 400125 |
| Mouse IgG1, κ Isotype Ctrl-PE (MOPC-21) | BioLegend (San Diego, CA, USA) | Cat #: 400111 |
| Mouse IgG1, κ Isotype Ctrl-BV510 (MOPC-21) | BioLegend (San Diego, CA, USA) | Cat #: 400171 |
| Mouse IgG1, κ Isotype Ctrl-PerCP/Cy5.5 (MOPC-21) | BioLegend (San Diego, CA, USA) | Cat #: 400149 |
| Rat IgG2a, κ Isotype Ctrl-PerCP/Cy5.5 (RTK2758) | BioLegend (San Diego, CA, USA) | Cat #: 400531 |
| Zombie Green™ Fixable Viability Kit | BioLegend (San Diego, CA, USA) | Cat #: 423111 |
| Cyto-Fast™ Fix/Perm Buffer Set | BioLegend (San Diego, CA, USA) | Cat #: 426803 |
| True-Nuclear™ Transcription Factor Buffer Set | BioLegend (San Diego, CA, USA) | Cat #: 424401 |
| CCL21 Mouse ELISA Kit | Abcam (Cambridge, United Kingdom) | Cat #: ab208985 |
| Treatment | Source | Identifier |
| InVivoPlus anti-mouse PD-1 | Bio X Cell (Lebanon, NH, USA) | Cat #: BP0146 |
| InVivoMAb anti-mouse 4-1BB (CD137) | Bio X Cell (Lebanon, NH, USA) | Cat #: BE0239 |
| InVivoPlus rat IgG2a isotype control | Bio X Cell (Lebanon, NH, USA) | Cat #: BP0089 |
| Reagent for DNA/RNA experiments | Source | Identifier |

| | | |
|---|---|---|
| psiCHECK™-2 Vectors | Promega (Madison, WI, USA) | Cat #: C8021 |
| pPACKH1-XL packaging mix | SBI (Palo Alto, CA, USA) | Cat #: LV510A-1 |
| Lipofectamine™ 3000 Transfection Reagent | Invitrogen (Waltham, MA, USA) | Cat #: L3000001 |
| mirVana™ miRNA Isolation Kit | Invitrogen (Waltham, MA, USA) | Cat #: AM1560 |
| miScript II RT Kit | Qiagen (Hilden, Germany) | Cat #: 218161 |
| LightCycler® 480 SYBR Green I Master | Roche (Penzberg, Germany) | Cat #: 04707516001 |
| Other Reagents | Source | Identifier |
| Growth factor reduced Matrigel | BD Biosciences (Franklin Lakes, NJ, USA) | Cat #: 354230 |
| ProLong™ gold antifade mountant | Invitrogen (Waltham, MA, USA) | Cat #: P36934 |
| AR6 buffer | PerkinElmer (Waltham, MA, USA) | Cat #: AR600250ML |
| Dynabeads™ Mouse DC (dendritic cell) enrichment | Invitrogen (Waltham, MA, USA) | Cat #: 11429D |
| CellXVivo Mouse Dendritic Cell Differentiation Kit | R&D Systems (Minneapolis, MN, USA) | Cat #: CDK008 |
| Mice | Source | Identifier |
| ACKR4-shRNA mice | Mirimus Inc (Brooklyn, NY, USA) | NA |
| C57BL/6 mice | The Charles River Laboratories (Wilmington, MA) | Stock No: 027 |
| Software | Source | Identifier |
| GraphPad Prism 8 | GraphPad Software (San Diego, CA, USA) | https://www.graphpad.com/ (accessed on 27 August 2021) |
| FlowJo v10.7.2 | BD Biosciences (Franklin Lakes, NJ, USA) | https://www.flowjo.com/ (accessed on 27 August 2021) |
| CIBERSORT | NA | https://cibersort.stanford.edu/ (accessed on 27 August 2021) |

© 2021 by the authors. Licensee MDPI, Basel, Switzerland. This article is an open access article distributed under the terms and conditions of the Creative Commons Attribution (CC BY) license (http://creativecommons.org/licenses/by/4.0/).
