# Supplementary material for: ACKR4 in Tumor Cells Regulates Dendritic Cell Migration to Tumor-Draining Lymph Nodes and T-Cell Priming
Source: Cancers (Basel). 2021 Oct 7;13(19):5021. doi: 10.3390/cancers13195021 (PMC8507805; doi:10.3390/cancers13195021)
Supplement: Supplementary file 1 [file cancers-13-05021-s001.zip › cancers-1377799-supplementary-final/Supplementary Figures/Figure S1.pdf]

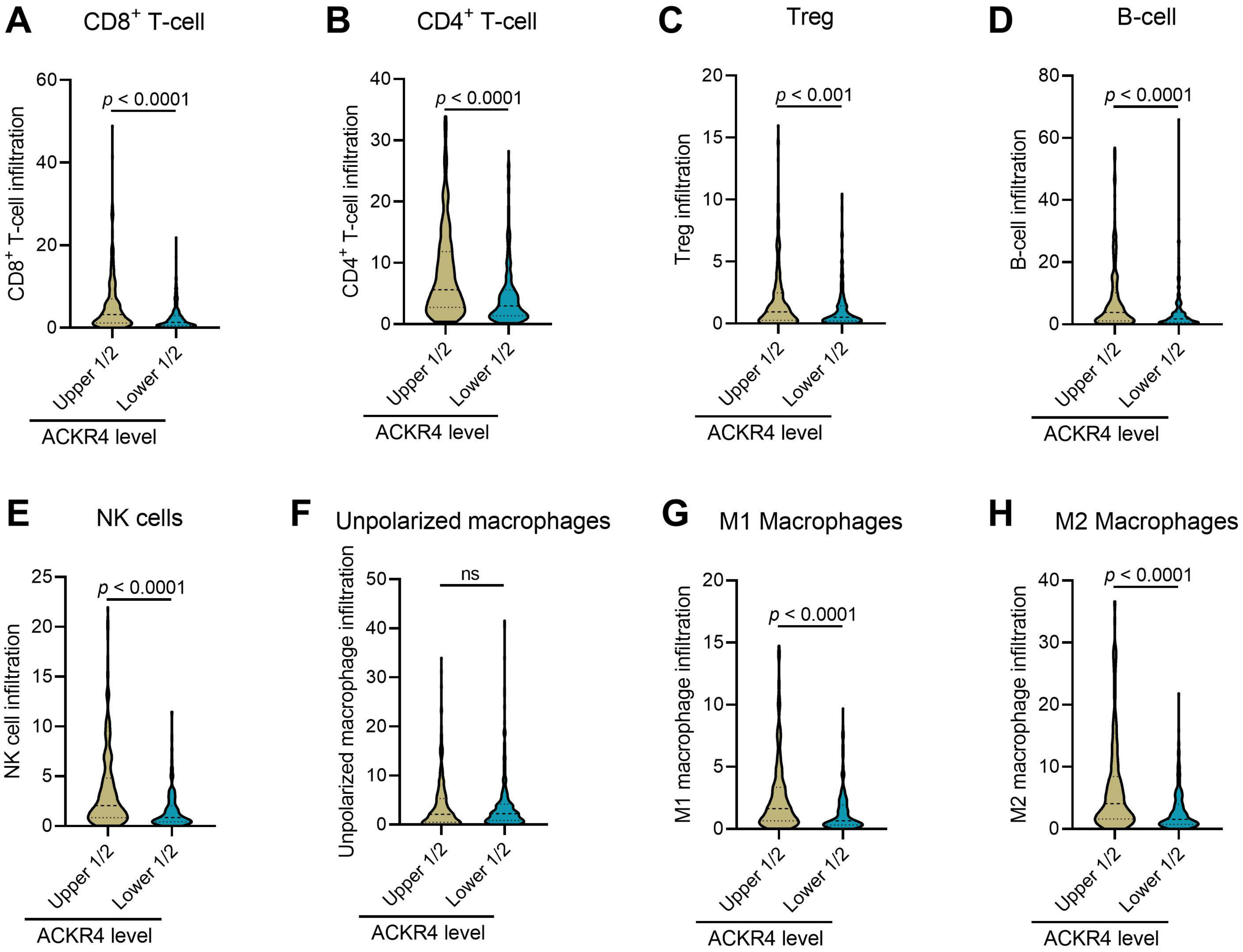

A
CD8+ T-cell
CD8+ T-cell infiltration
p < 0.0001
Upper 1/2
Lower 1/2
ACKR4 level
B
CD4+ T-cell
CD4+ T-cell infiltration
p < 0.0001
C
Treg
Treg infiltration
p < 0.001
D
B-cell
B-cell infiltration
p < 0.0001
E
NK cells
NK cell infiltration
p < 0.0001
F
Unpolarized macrophages
Unpolarized macrophage infiltration
ns
G
M1 Macrophages
M1 macrophage infiltration
p < 0.0001
H
M2 Macrophages
M2 macrophage infiltration
p < 0.0001
