# Supplementary figures and images for: ACKR4 in Tumor Cells Regulates Dendritic Cell Migration to Tumor-Draining Lymph Nodes and T-Cell Priming
Source: Cancers (Basel). 2021 Oct 7;13(19):5021. doi: 10.3390/cancers13195021 (PMC8507805; doi:10.3390/cancers13195021)

**A**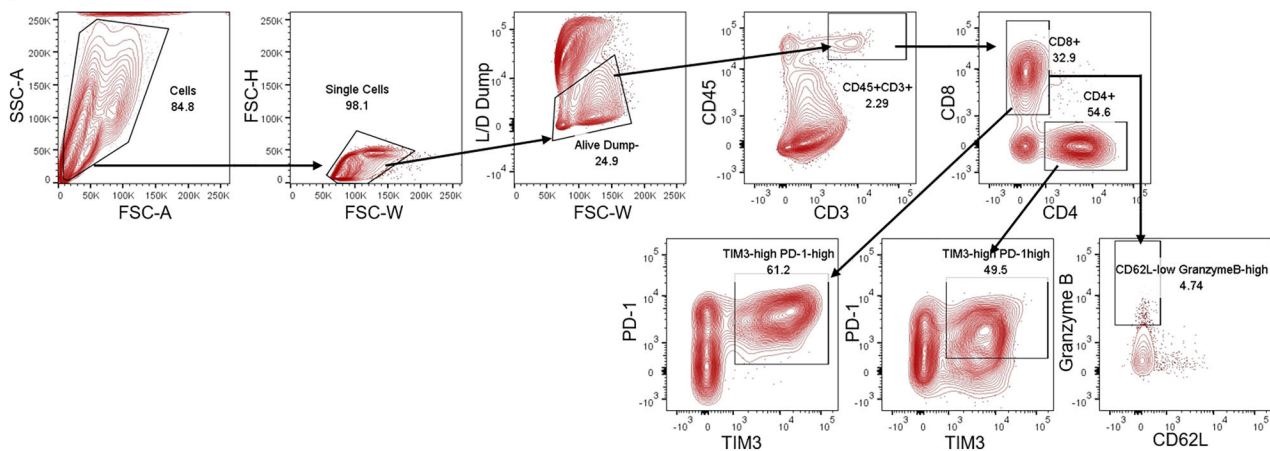**B**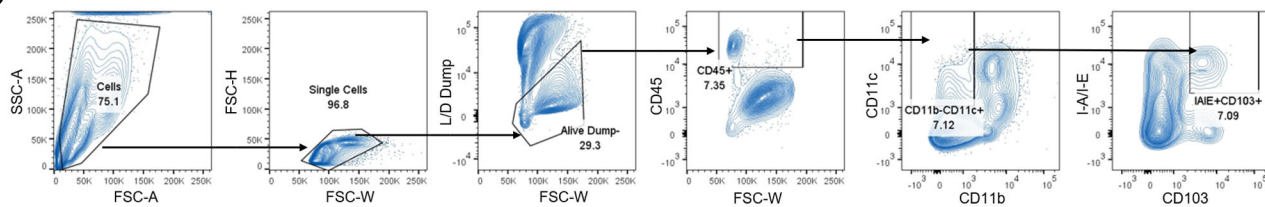**C**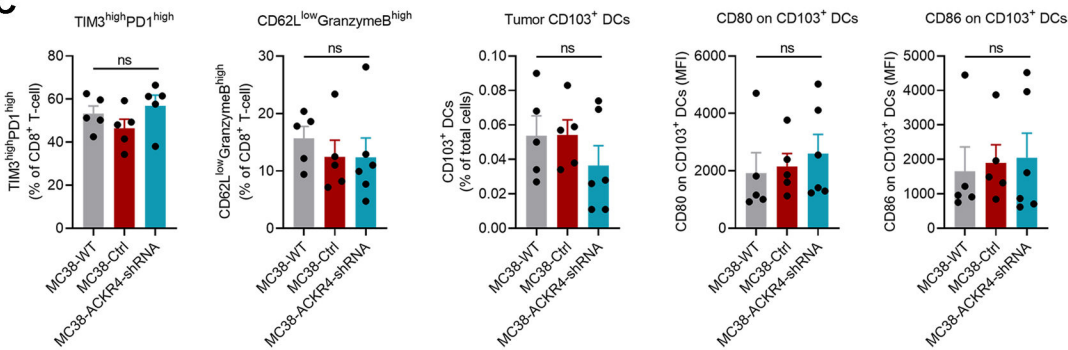**D**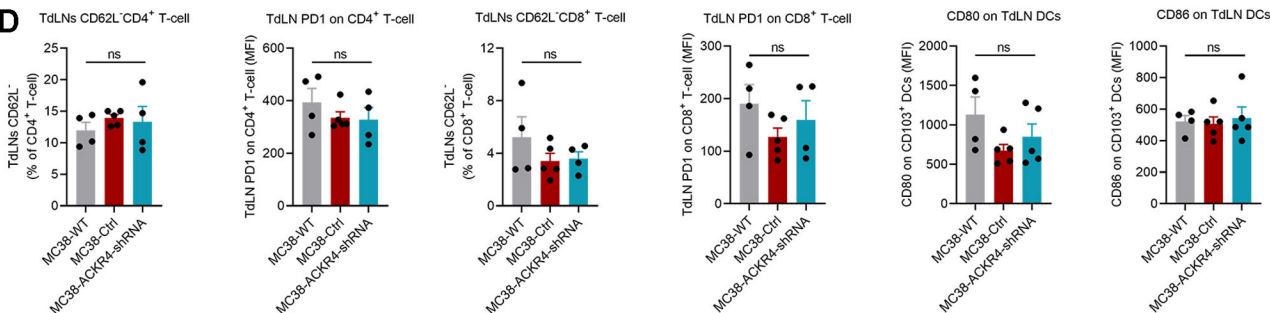

Supplement: Supplementary file 1 [file cancers-13-05021-s001.zip › cancers-1377799-supplementary-final/Supplementary Figures/Figure S2.pdf]

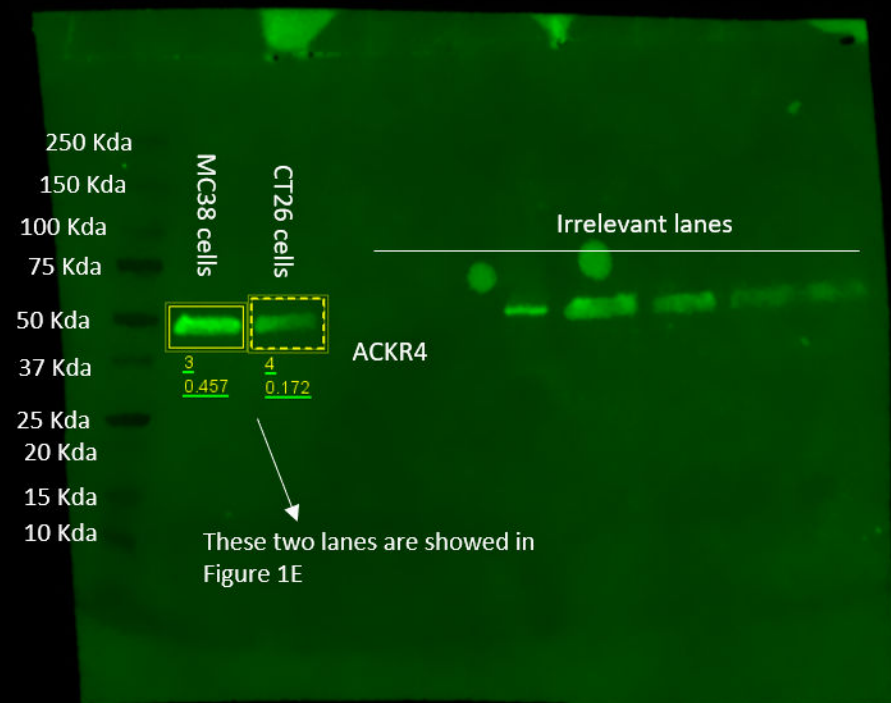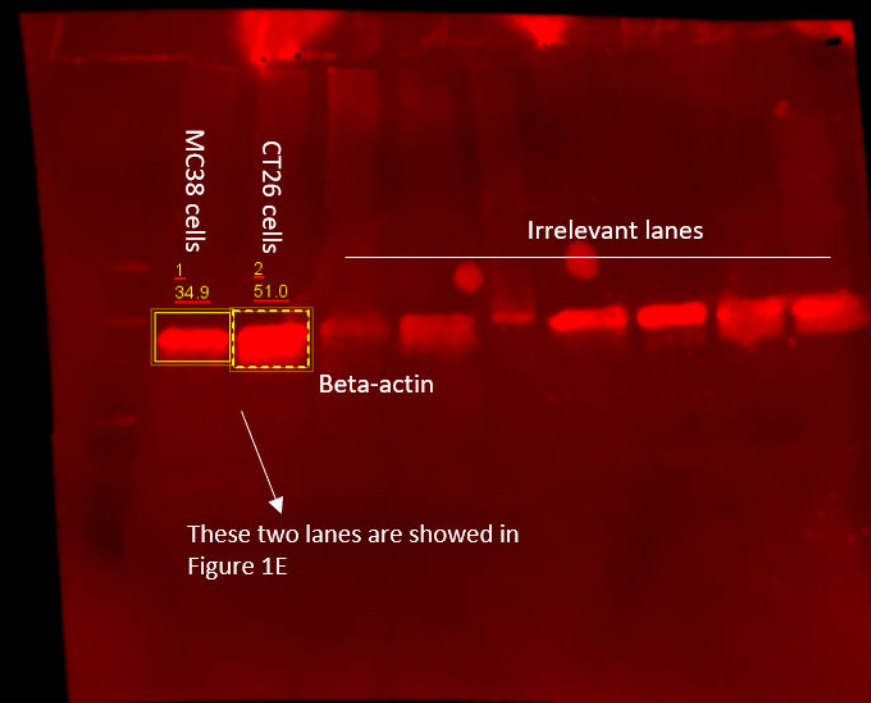

Supplement: Supplementary file 1 [file cancers-13-05021-s001.zip › cancers-1377799-supplementary-final/Supplementary Figures/Figure S3.pdf]

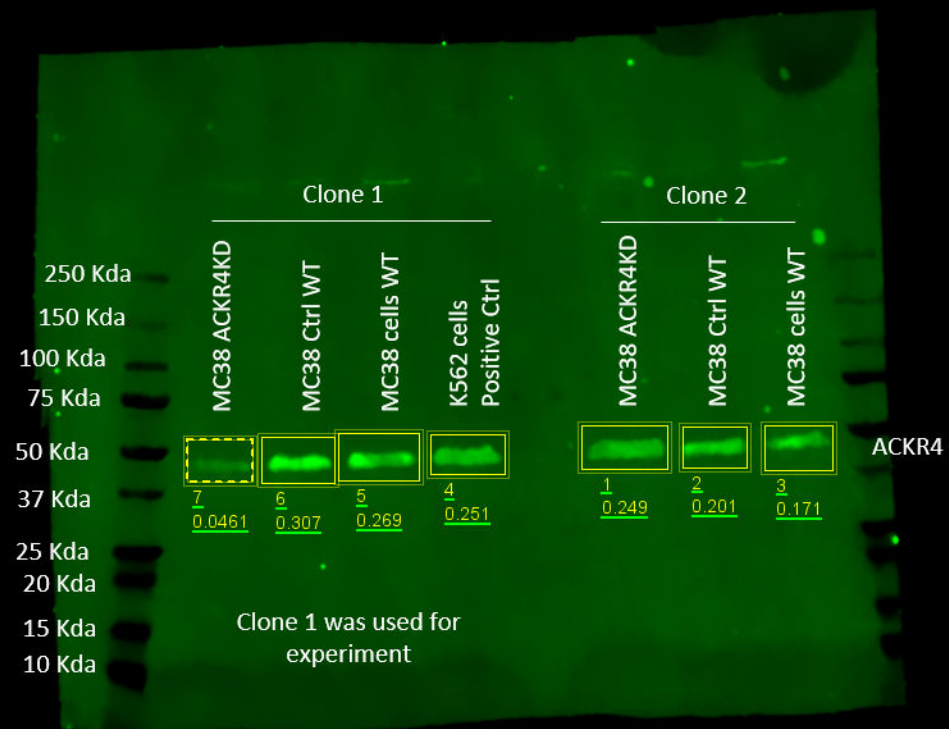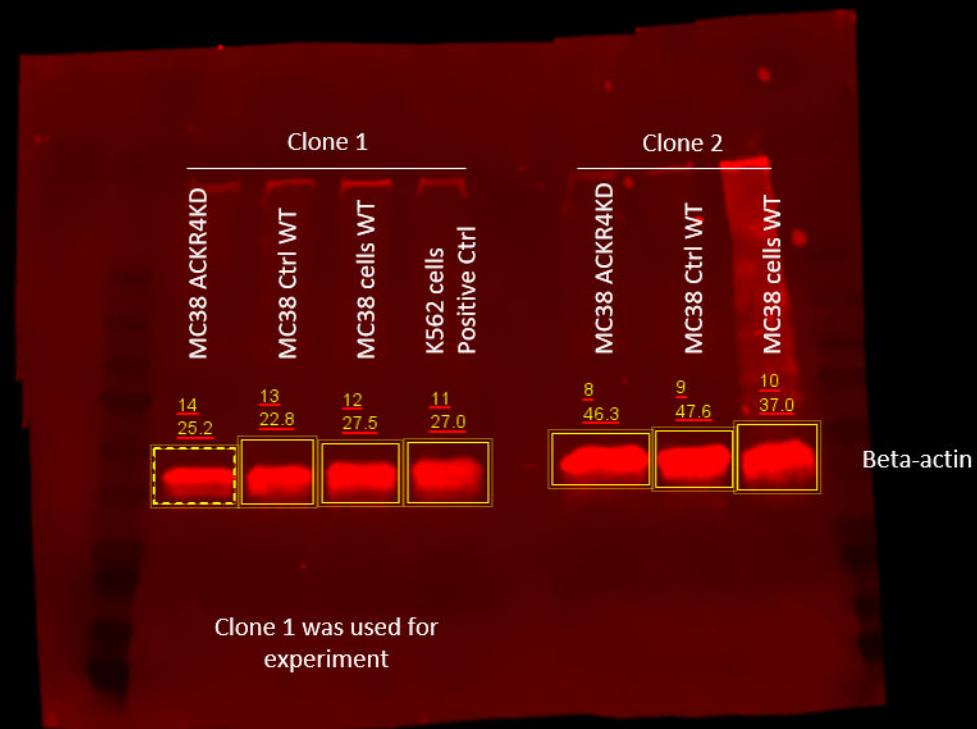

Supplement: Supplementary file 1 [file cancers-13-05021-s001.zip › cancers-1377799-supplementary-final/Supplementary Figures/Figure S4.pdf]

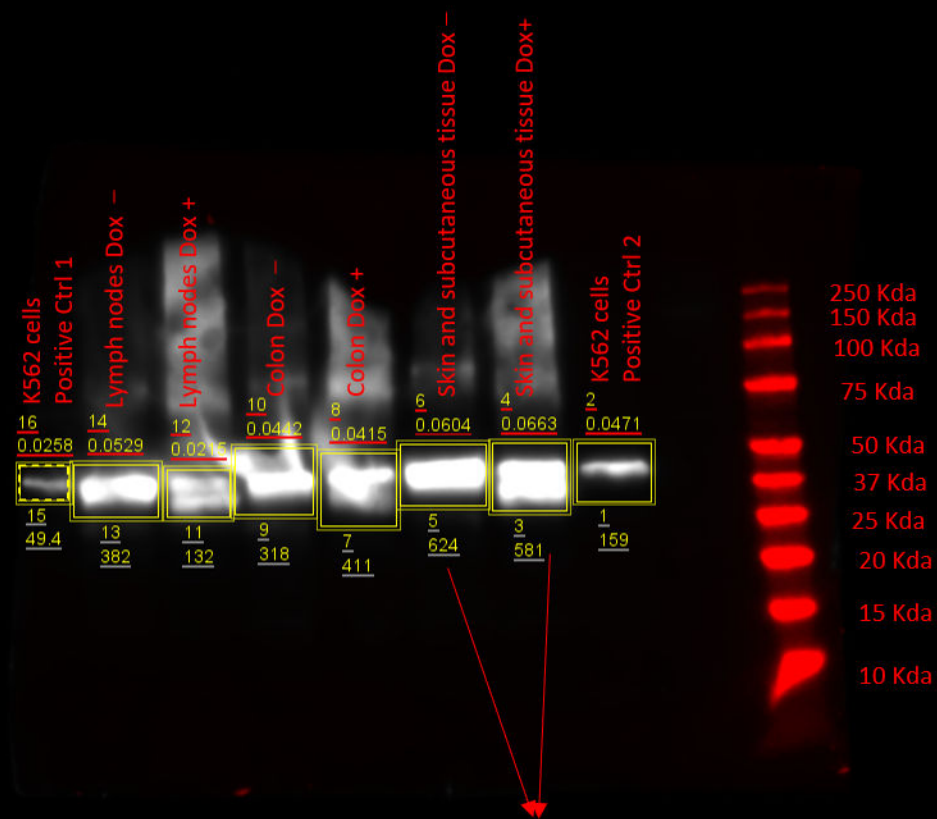

These two lanes are showed in Figure 2C

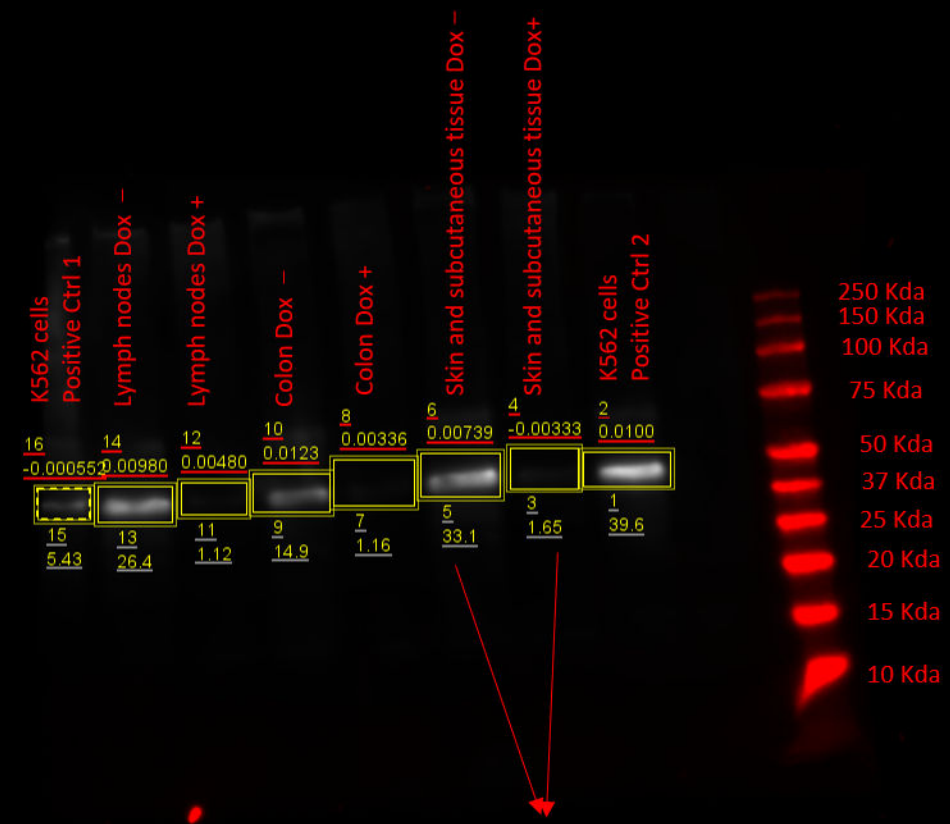

These two lanes are showed in Figure 2C

Supplement: Supplementary file 1 [file cancers-13-05021-s001.zip › cancers-1377799-supplementary-final/Supplementary Figures/Figure S5.pdf]
